# Kinetic study of H-terminated silicon nanowires oxidation in very first stages

**DOI:** 10.1186/1556-276X-8-41

**Published:** 2013-01-21

**Authors:** Muhammad Y Bashouti, Kasra Sardashti, Jürgen Ristein, Silke Christiansen

**Affiliations:** 1 Max Planck Institute for the Science of Light Physics department Günther-Scharowsky-St. 1, Erlangen, 91058, Germany; 2 Technical Physics, University of Erlangen-Nürnberg, Erwin-Rommel St.1, Erlangen, 91058, Germany

**Keywords:** Silicon nanowires, Oxidation, Kinetics, Activation energy

## Abstract

Oxidation of silicon nanowires (Si NWs) is an undesirable phenomenon that has a detrimental effect on their electronic properties. To prevent oxidation of Si NWs, a deeper understanding of the oxidation reaction kinetics is necessary. In the current work, we study the oxidation kinetics of hydrogen-terminated Si NWs (H-Si NWs) as the starting surfaces for molecular functionalization of Si surfaces. H-Si NWs of 85-nm average diameter were annealed at various temperatures from 50°C to 400°C, in short-time spans ranging from 5 to 60 min. At high temperatures (*T* ≥ 200°C), oxidation was found to be dominated by the oxide growth site formation (made up of silicon suboxides) and subsequent silicon oxide self-limitation. Si-Si backbond oxidation and Si-H surface bond propagation dominated the process at lower temperatures (*T* < 200°C).

## Background

During the last decade, silicon nanowires (Si NWs) have been studied extensively to be employed in the modern electronic industry in the direction of the size reduction and efficiency boost of the devices [1]. Because of the high surface to volume ratio, Si NWs’ properties depend firmly on their surface conditions and surface terminations, in particular. The oxidation of Si NWs, when exposed to ambient air, is believed to have a detrimental effect on their electrical properties due to the low quality of the oxide, giving rise to the uncontrolled interface states and enhanced carrier recombination rates [2]. This necessitates protection of Si NWs’ surfaces against oxidation via termination by various chemical moieties (i.e., alkyls and alkenyls) [3,4]. However, to better prevent oxide formation, a deeper understanding of the Si NW’s oxidation mechanisms and kinetics is essential. For planar Si, the widely known Deal-Grove (DG) model considers the interfacial oxidation reaction and oxidant diffusion as the major rate-determining reaction steps for short and long oxidation times, respectively [5]. DG model has undergone a number of modifications due to imprecise prediction of the oxidation behavior at low temperatures (*T* ≤ 700°C) in convex/concave surfaces and for very thin oxide layers [6-8]. Specifically, in sufficiently small Si NWs (*d* ≤ 44 nm), oxidation can be completely retarded by the compressive stress normal to the oxide/NW interface [9,10]. Nevertheless, the studies on the oxidation mechanisms of Si NWs have been focused mostly on the formation of thick oxide layers at relatively high temperatures and long times, overlooking the early stages of oxidation which involve removal of surface functionalities and suboxides formation.

In this article, thermal stability of hydrogen-terminated Si NWs of 85-nm average diameter was investigated by means of the surface-sensitive X-ray photoelectron spectroscopy (XPS) for a variety of temperatures and times. H-terminated surfaces are of importance since they are considered as the starting surfaces for further functionalization of Si NWs [11-15]. The different kinetic behavior for the three transient silicon suboxides and SiO_2_ has been shown. Growth regimes were mainly addressed by four different phenomena including Si-Si backbond oxidation, surface bond propagation, suboxide growth site formation, and self-limited oxidant diffusion. A preliminary oxidation mechanism, elucidating the influence of time and temperature on the role of latter factors, is outlined.

## Methods

### Synthesis of initial Si NWs

To produce Si NWs, the vapor–liquid-solid (VLS) technique for silane (SiH4) gas, assisted by gold (Au) as silane decomposition catalyst, was employed. Prior to the VLS process, the native oxides on substrates of Si(111) have to be removed through etching in diluted HF. A thin gold layer of 2 nm in thickness was then sputtered on the etched substrates. After being transferred to the VLS operation chamber, the substrates were subjected to temperature and pressure of ≈580°C and ≈ 5 × 10^−7^ mbar for 10 min, as to be annealed. Subsequently, to grow nanowires on the surface, temperature was reduced to ≈520°C and a gas mixture of 5 to 10 ccm (standard cm^3^ min^−1^) Ar and 5 ccm SiH4 was introduced for 20 min at a pressure ranging from 0.5 to 2 mbar.

### Si NWs hydrogen termination

The grown Si NWs has to be treated on their surface. Si NW were first cleaned by N_2_(g) flow for several seconds and then exposed in a sequence to buffered HF solution (pH = 5) and NH_4_F (40% in water) for 30 to 50 s and 30 to −180 s, respectively. H-terminated Si NWs were rinsed by water for less than 10 s per side to prevent the oxidation and dried in N_2_(g) for 10 s.

### Oxide growth in Si NWs

To evaluate the thermal stability of hydrogen atoms bonded to NWs’ surfaces and find dominant oxidation mechanisms, H-Si NWs were annealed at atmospheric condition in six distinct temperatures of 50°C, 75°C, 150°C, 200°C, 300°C, and 400°C, each for five different time-spans: 5, 10, 20, 30, and 60 min. The annealing and hydrogen-termination processes were gentle in the sense that they did not melt the Si NWs or change their diameters.

### Characterization of Si NWs

Pristine Si NWs were examined by scanning electron microscopy (SEM, Toshiba S-4800, Toshiba International (Europe) Ltd., Uxbridge, UK) with 5.0 kV voltage and 10.0 μA current, on top and side views. After each heating stage, the specimens were scanned by home-made XPS. Core level and valance band photoelectron spectra were excited by monochromatic Al K radiation (1,487 eV) and collected, at take-off angle of 35°, by a hemispherical analyzer with adjustable overall resolution between 0.8 and 1.2 eV. The surveys were conducted in various ranges of electron energies including the overall binding energy survey (0 to 1,000 eV) besides individual spectra for Si 2*p* (95.0 to 110.0 eV), C 1 *s* (282.0 to 287.0 eV) and O 1 *s* (520 to 550 eV) which were monitored more accurately in a discrete number of scans. All spectra were taken at room temperature in a UHV chamber of about 10^−10^ Torr pressure. The resulting XPS spectra were analyzed by spectral decomposition using the XPS peak software and their oxide levels were determined.

## Results and discussion

**Figure 1:**
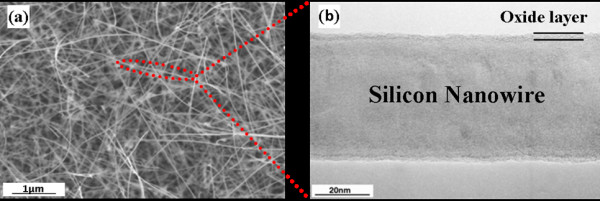
**SEM and transmission electron microscopy (TEM) micrographs of the pristine Si NWs.** (**a**) Top-view SEM micrograph of the Si NWs grown by VLS mechanism showing their random orientation. (**b**) TEM image of an individual Si NW cross-section representing the continuous native oxide layer of 3 to 4 nm in diameter atop. Regarding the micrographs, the Si core diameter can be estimated as 50 ± 10 nm. The red dotted line insists on the fact that TEM micrograph is taken for a single Si NW among the large ensemble observed through SEM.

The VLS-grown Si NWs used in this study were randomly oriented with average diameter and length of 84.96 nm and 3.508 μm, respectively. The pristine Si NWs are covered by a native oxide layer of 1 to 4 nm. SEM and transmission electron microscopy (TEM) micrographs of the pristine Si NWs are depicted in Figure 1. Residual gold nanoparticles were removed by rinsing the Si NWs into HNO_3_ solution preventing its catalytic effect on oxidation.

**Figure 2:**
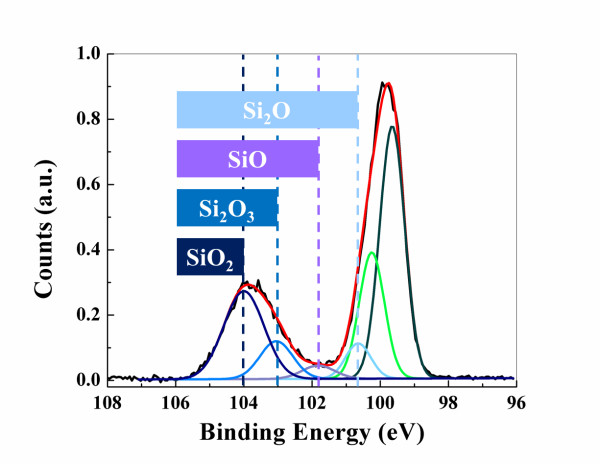
**Spectral decomposition of Si 2*p *spectrum of Si NWs sample annealed at 500°C for 60 min.** Spectral decomposition of Si 2*p* spectrum of Si NWs sample annealed at 500°C for 60 min, having all the relevant suboxide and silicon peaks (Si 2*p*3/2 in dark green and Si 2*p*1/2 in light green). The black line is the original spectrum, while the red graph represents the fitting curve which is sum of all of the decomposed peaks and fit well the experimentally obtained spectrum.

As an illustrative Si 2*p* spectrum of oxidized Si NWs, the Si 2*p* spectrum of the H-terminated Si NWs annealed at 500°C for 60 min is depicted in Figure 2. By formation of even very thin silicon dioxide layers, the Si 2*p* XPS survey of Si NWs changes, showing a peak between the binding energies of 102 to 104 eV. To quantitatively evaluate the oxidation process, Si 2*p* spectral decomposition was conducted on the spectra after Shirley background subtraction, through a curve-fitting procedure using Gaussian-Lorentzian functions [16]. Consequently, the Si 2*p* spectra can be divided into six different sub-peaks including two silicon spin-splitting peaks as Si 2*p*_1/2_ and Si 2*p*_3/2_, three silicon sub-stoichiometric oxides (known as suboxides) peaks as Si_2_O, SiO and Si_2_O_3_, and the silicon dioxide (SiO_2_) peak. The chemical shifts (Δ) of the sub-peaks obtained in Figure 2 relative to the Si 2*p*_3/2_ (at 99.60 ± 0.02 eV) are as follows: Si 2*p*_1/2_ (Δ = 0.60 eV), Si_2_O (Δ = 0.97 eV), SiO (Δ = 1.77 eV), Si_2_O_3_ (Δ = 2.50 eV), and SiO_2_ (Δ = 3.87 eV).

The amount of each of suboxides, relative to the amount of intact silicon, can be calculated by dividing the integrated area under the suboxide’s peak (*A*_SiOx_) by the sum of the integrated area under Si 2*p*_1/2_ and Si 2*p*_3/2_ peaks (*A*_Si 2*p*1/2_ + *A*_Si 2*p*3/2_). The resulting value is called suboxide intensity, shown by *I*_SiOx_. In addition, total oxide intensity (*I*_ox_) can be calculated as the sum of all the four suboxide intensities (*I*_ox_ = *I*_Si2O_ + *I*_SiO_ + *I*_Si2O3_ + *I*_SiO2_). Oxide intensity can also be expressed in number of monolayers, regarding the fact that each 0.21 of oxide intensity corresponds to one oxide monolayer [17]. The total oxide intensity, besides suboxide intensities for the Si NWs specimens annealed at 150°C and 400°C, is listed in Table 1. Except SiO_2_, all the suboxide intensities for both of the annealing temperatures are comparable and more or less show very slight variations over the annealing time. However, at 150°C, suboxides hold a larger share of the total oxide intensity whereas at 400°C, SiO_2_ mainly contributes to the overall oxide amount detected.

**Table 1:** **Intensity of the silicon suboxides for the samples annealed at 150**°**C and 400**°**C**

	***T*** = **150**°**C**					***T*** = **400**°**C**				
**Intensity/oxidation time (min)**	**5**	**10**	**20**	**30**	**60**	**5**	**10**	**20**	**30**	**60**
Si_2_O	0.317	0.269	0.252	0.289	0.198	0.235	0.227	0.186	0.212	0.249
SiO	0.067	0.092	0.102	0.151	0.148	0.107	0.089	0.142	0.095	0.104
Si_2_O_3_	0.026	0.078	0.076	0.126	0.088	0.157	0.077	0.149	0.139	0.083
SiO_2_	0.228	0.350	0.414	0.666	0.787	1.181	1.390	1.569	1.604	1.922
Total	0.640	0.790	0.845	1.234	1.223	1.680	1.785	2.047	2.052	2.360

**Figure 3:**
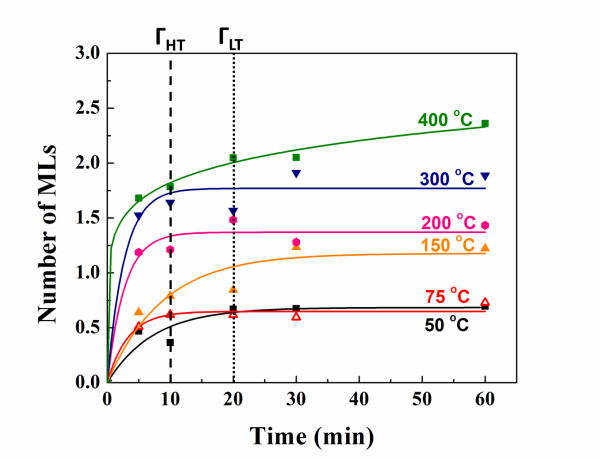
**Variation of total oxide monolayer over time for the six different oxidation temperatures.** The two dashed and dotted lines represent saturation times (Γ) for high- and low-temperature oxidation, respectively.

Variation in the total oxide intensity (*I*_ox_) for all the six temperatures over oxidation time up to 60 min is shown in Figure 3. For both the high temperature (*T* ≥ 200°C) and low-temperature oxidation (*T* < 200°C), the oxide intensity reaches a saturation level beyond which the oxide amount grows negligibly. However, in low-temperature oxidation, the time to reach 80% of the saturation levels (defined as Γsat) is in the range of 20 to 30 min, whereas in high-temperature oxidation it ranges from 8 min to 12 min. Average Γsat for high- and low-temperature oxidation are marked in Figure 3 by dashed and dotted lines, respectively. This indicates roughly both similarities and differences between the underlying oxidation mechanisms in these two temperature ranges. The presence of the saturation levels reveals the fact that a mechanism is hindering further oxide growth after formation of a certain oxide level. On the other hand, the growth rates differ between the two temperature ranges, revealing the existence of mechanisms with different thermal activation energies.

The growth of oxide in planar silicon in thick layers and at high temperatures has been successfully expressed by the Deal-Grove model. However, it breaks down in very thin oxide layers and has been modified considering the suboxides as nucleation sites (or oxide growth sites) that are necessary for oxide build-up [6]. Through high-temperature oxidation, silicon suboxides exhibit relatively constant values after a sharp increase in their intensities. Therefore, in the early stages of Si NWs oxidation, formation of the growth sites composed of suboxides can be taken into account as the major mechanism.

**Figure 4:**
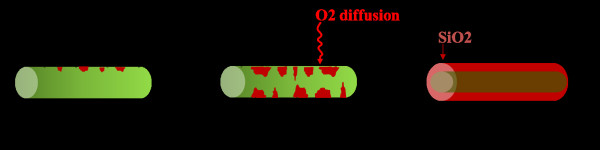
**Scheme of the suggested mechanism for high**-**temperature oxidation of the H**-**terminated Si NWs.**

Further oxidation and rise of the flat tail indicate existence of a second mechanism, which is impeding oxide formation at the suboxide growth sites. In Si NWs, such retarded oxidation behaviors have mostly been attributed to their geometry and presence of compressive stresses normal to the silicon/silicon oxide interfaces that limit further oxide growth and its expansion [8,10]. Nevertheless, compressive stresses are more expected for NWs of diameter below 44 nm which is far below the average diameter of the Si NWs studied here [9]. Additionally, comparison between Si NWs and planar Si(100) oxidation behavior in the same time and temperature ranges showed similar flat tails of oxide [18]. Therefore, the retarded oxidation in Si NWs, in analogy with planar silicon, can be attributed to the self-limited oxidation caused by the act of firstly formed oxide layer as a diffusion barrier [19]. The two mechanisms are summarized in Figure 4.

**Figure 5:**
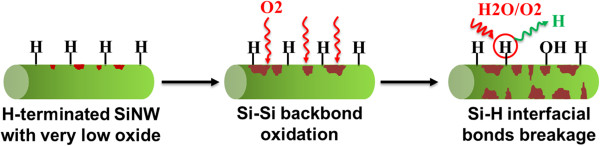
**Scheme of the suggested mechanism for low**-**temperature oxidation of the H**-**terminated Si NWs.**

At lower temperatures, increase of the total oxide intensity is accompanied by the rise in the intensity of suboxides with amounts comparable to SiO_2_ intensity (Table 1). Backbond oxidation can be considered as the primary mechanism causing formation Si-O-Si bonds below the surface-terminating Si-H bonds. The backbonds can be oxidized in different oxidation states and can finally form the full oxide layer atop. Compared to planar samples, Si NWs exhibit faster backbond oxidation, indicating the effect of circumferential tensile stresses on the stability of Si-Si bonds [18]. For longer oxidation times, upon formation of a larger number of oxidized backbonds, isolated Si-OH bonds start to form upon interaction of Si-H and Si-O bonds in the oxidized backbond [20]. By completion of the backbond oxidation, besides the Si-OH formation, remaining Si-H surface bonds start to rupture and hydrogen propagation begins. Low-temperature oxidation mechanism is summarized in the scheme illustrated in Figure 5. It should be noted that the hydroxyl groups shown in Figure 5 represent both the isolated hydroxyl groups formed throughout the oxidation and after completion of oxidation as frequent for SiO_2_ in H_2_O-containing environments [21].

## Conclusions

In conclusion, the growth kinetics of the suboxides and silicon dioxide is highly dependent to temperature and time. At lower temperatures, oxidation is first controlled by backbond oxidation. After full oxidation of the backbonds, Si-H bond rupture dominates the process kinetics. At higher temperatures, suboxide nucleation sites (known as oxide growth sites) control the early stages of oxidation. After complete formation of the very first oxide monolayers, further oxidation is self-limited as the oxidant’s diffusion through the oxide layers is impaired. These findings suggest a perspective on more efficient methods to stabilize Si NWs against oxidation over the long term.

## Abbreviations

SEM: Scanning electron microscopy; Si NWs: Silicon nanowires; VLS: Vapor–liquid-solid; XPS: X-ray photoelectron spectroscopy.

## Competing interests

The authors declare that they have no competing interests.

## Authors’ contributions

MYB and KS carried out the experiments and wrote the article. JR and SHC conceived of the study and participated in its design and coordination. All authors read and approved the final manuscript.
